# The Effects of Smoking on Ultrasonographic Thickness and Elastosonographic Strain Ratio Measurements of Distal Femoral Cartilage

**DOI:** 10.3390/ijerph13040434

**Published:** 2016-04-21

**Authors:** Harun R. Gungor, Kadir Agladioglu, Nuray Akkaya, Semih Akkaya, Nusret Ok, Levent Ozçakar

**Affiliations:** 1 Orthopedics and Traumatology Department, Medical Faculty, Pamukkale University, Denizli 20070, Turkey; semihakkaya@yahoo.com (S.A.); oknusret@gmail.com (N.O.); 2 Radiology Department, Medical Faculty, Pamukkale University, Denizli 20070, Turkey; kadiragladi@yahoo.com; 3 Physical and Rehabilitation Medicine Department, Medical Faculty, Pamukkale University, Denizli 20070, Turkey; nrakkaya@gmail.com; 4 Physical and Rehabilitation Medicine Department, Medical Faculty, Hacettepe University, Ankara 06100, Turkey; lozcakar@yahoo.com

**Keywords:** distal femur, cartilage, smoking, ultrasound, sonoelastography

## Abstract

Although adverse effects of smoking on bone health are all well known, data on how smoking interacts with cartilage structure in otherwise healthy individuals remains conflicting. Here, we ascertain the effects of cigarette smoking on sonoelastographic properties of distal femoral cartilage in asymptomatic adults. Demographic characteristics and smoking habits (packets/year) of healthy volunteers were recorded. Medial, intercondylar, and lateral distal femoral cartilage thicknesses and strain ratios on the dominant extremity were measured with ultrasonography (US) and real time US elastography. A total of 88 subjects (71 M, 17 F; aged 18–56 years, *N* = 43 smokers and *N* = 45 nonsmokers) were evaluated. Mean amount of cigarette smoking was 10.3 ± 8.9 (1–45) packets/year. Medial, intercondylar and lateral cartilage were thicker in smokers than nonsmokers (*p* = 0.002, *p* = 0.017, and *p* = 0.004, respectively). Medial distal femoral cartilage strain ratio was lower in smokers (*p* = 0.003). The amount of smoking was positively correlated with cartilage thicknesses and negatively correlated with medial cartilage strain ratios (*p* < 0.05). Femoral cartilage is thicker in smokers but has less strain ratio representing harder cartilage on the medial side. Future studies are needed to understand how these structural changes in the knee cartilage should be interpreted with regard to the development of knee osteoarthritis in smokers.

## 1. Introduction

The relationship between cigarette smoking and chronic musculoskeletal system disorders (e.g., degenerative disc disease) is a well-known entity in the literature [1,2]. However, although an inverse correlation has been reported between smoking and knee osteoarthritis, the effects of smoking on knee cartilage remains conflicting [3,4]. Yet, while some research suggests protective effects of smoking on osteoarthritis [4,5], others propose that smoking increases cartilage destruction [6]. Previously proposed mechanisms for the protective effect of nicotine on cartilage in an early osteoarthritis rat model were reduction of the serum level of TNF-α, reduction of the expression of TNF-α in the synovial tissue, and increase in the expression of α7nAChR in the synovial tissue [7]. On the other hand, it has been shown that smoking and nicotine delay revascularization of bone grafts, increase pseudo-arthrosis risk in spinal fusion operations, and inhibit radiographic and biomechanical bone healing in rabbits [8]. Herewith, data on how smoking interacts with cartilage structure of asymptomatic individuals is insufficient.

Ultrasound (US) is reported as a reliable and easily accessible imaging method for measurement of cartilage thickness [9,10,11,12]. Sonoelastography is a relatively new US technique to objectively evaluate tissue elasticity [13,14]. In a recent study, it has been reported that healthy and pathologic cartilage can be distinguished by measuring strain ratios using real-time sonoelastography [15]. To the best of our knowledge, distal femoral cartilage has not been evaluated for the effects of cigarette smoking in this regard. As such, we tried to explore the effects of cigarette smoking on sonoelastographic properties of distal femoral cartilage in asymptomatic adults.

## 2. Materials and Methods

### 2.1. Participants

This study was designed as a cross-sectional research. Healthy volunteers older than 18 years of age were enrolled in the study. Non-smoking group comprised subjects (*N* = 45) who have never smoked in their lives. Smoking group comprised subjects (*N* = 43) who uninterruptedly smoked (≥1 packets/day) at least in the last 12 months, and smoking amount was calculated as packets/year (number of cigarette per day × smoking year). All subjects gave their informed consent for inclusion before they participated in the study. The study was conducted in accordance with the Declaration of Helsinki, and the protocol was approved by the Ethics Committee of Pamukkale University Medical Faculty (No: 60116787-020/33103). 

Exclusion criteria were the presence of rheumatoid arthritis, crystal arthropathy, reactive arthritis or major psoriasis, restricted knee range of motion, lower limb mechanical axis deviation, hip arthritis and history of knee infection, operation, fracture, ligamentous or meniscal injury, and osteochondral lesion.

Demographic and clinical characteristics of the subjects were recorded including age, weight, height, body mass index (BMI- kg/m^2^), educational status, occupation, dominant extremity, and cigarette smoking status (packets/year). Knee pain during rest and activity were evaluated using visual analogue scale (VAS) whereby 0 refers to “no pain” and 10 to “the worst pain”. VAS scores were used to further evaluate the volunteers whether they meet the inclusion criteria. Tegner activity scores were recorded between 1 and 9 for all subjects to compare the activity level of smoker and non-smoker groups [16]. Demographical characteristics and smoking habits of participants were recorded according to the self-reported information from the volunteers by one of the authors (H.R.G.). Clinical examinations of the volunteers for existence of musculoskeletal findings (existence of pain with range of motion, general musculoskeletal examination for possible deformities) were performed, and characteristics were recorded by another author (N.O.) who was blinded to participants’ smoking habits and ultrasonographic evaluation results. Ultrasonographic imaging and measurements were performed by the radiologist (K.A.) who was also blinded to participants’ smoking habits.

### 2.2. Ultrasonographic and Sonoelastographic Evaluations

**Figure 1:**
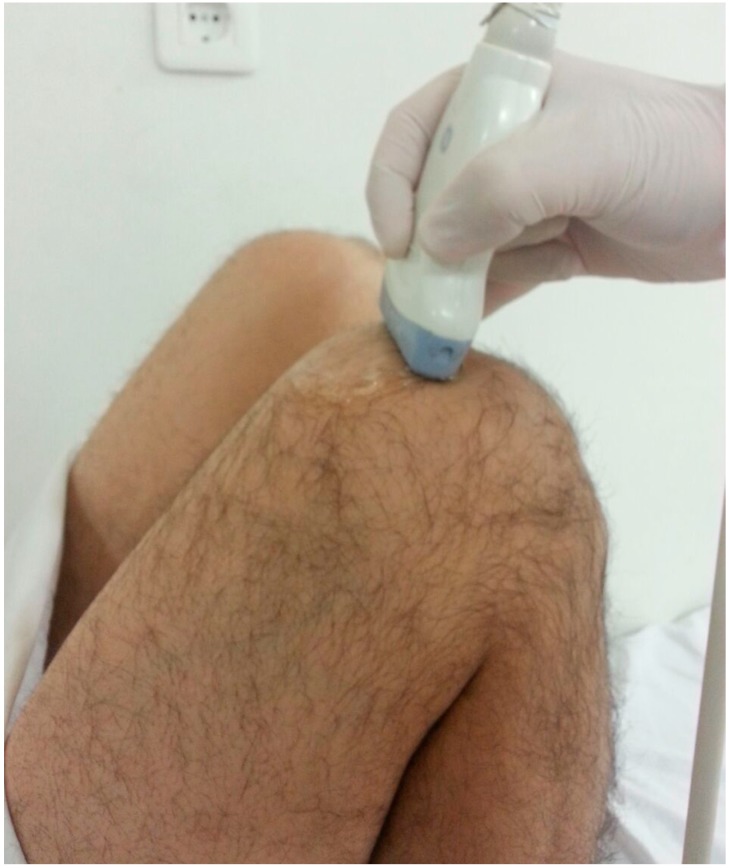
Experimental setup position of the knee and the ultrasound probe.

US examinations were performed using a 2.5–13 MHz linear probe (Logiq E9, GE Medical Systems, Wauwatosa, WI, USA). Subjects lay supine on the examination table with maximum flexion (>125°) of the dominant knee (Figure 1) [17]. The same sonographer (KA) who was blinded to the subjects’ data performed all the ultrasonographic and sonoelastographic examinations. During axial imaging, cartilage thicknesses were measured from the lateral condyle, intercondylar area, and the medial condyle. Measurements were taken from the midpoints of the aforementioned three areas by drawing a vertical line between cartilage-bone and synovial space-cartilage surfaces [18]. Three successive measurements were performed for each site, and the mean values were recorded. For intra-observer reliability, measurements were repeated in two sessions with intervals of at least two hours.

**Figure 2:**
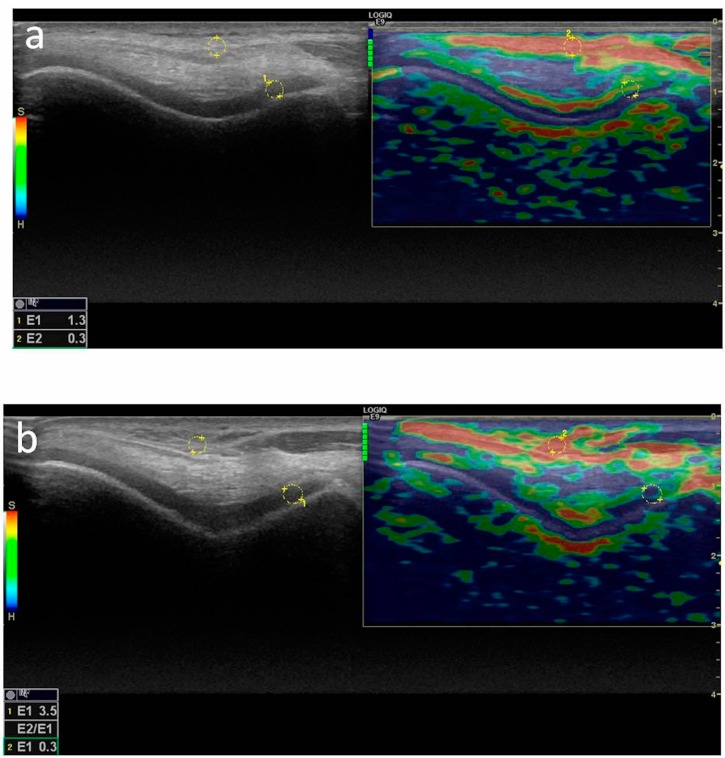
Color-coded real time images of distal femoral cartilage of non-smoking (**a**) and smoking (**b**) volunteers.

During sonoelastography, the US probe was placed on the distal femoral cartilage of the dominant knee, while subjects were kept in the same position as described above. Compression with slight vibrating motions (rhythmic compression and relaxation cycles) was applied to acquire the appropriate images. Color scale was evaluated on the US monitor (at least five green columns were obtained out of seven) to maintain the relevance and the standardization of the applied compression. Color-coded real time images were recorded on B-mode screen and the machine’s software coded colors in a scale of 1–6, between red (soft) and blue (hard) (Figure 2). Three recorded US images were chosen randomly, and circular areas—regions of interest (ROI)—were marked on the reference (subcutaneous) tissue and the three distal femoral cartilage regions (midpoints of medial, intercondylar, and lateral distal femur that were designated as cartilage on gray scale images). Again, the machine’s software calculated the strain ratios (reference tissue strain/cartilage strain) of each region. All ROIs marked on both reference tissue and distal femoral cartilage regions had the same size. Three successive measurements were performed, and the mean values were recorded [15,19]. Decreases in strain ratio indicate a hardening of cartilage tissue compared to reference tissue.

### 2.3. Statistical Analysis

The data were analyzed with Statistical Package for Social Sciences software (SPSS Version 17, Chicago, IL, USA). Descriptive statistics were expressed as mean ± standard deviation, frequency, and percentage. Mean values of the groups were compared using Student’s *t*-test, and categorical variables were compared using a chi-square test. Pearson’s coefficient was used to analyze correlations between smoking amount and US parameters. Statistical significance was set at *p* < 0.05. Interclass correlation coefficient (ICC with confidence interval 95%) was used for intra-observer reliability of the US measurements for the mean values of two successive measurements. *A priori* power analysis proved that at least 43 volunteers were needed in each group for a power of 80% (β = 0.20, α = 0.05). An *a posteriori* power analysis referring to the number of individuals in each group for each variable (mean ± SD) given on Table 1 with a statistically significant difference between smokers and non-smokers showed 92.5% power according to results of medial distal femoral cartilage thickness, 76.3% power according to results of intercondylar distal femoral cartilage thickness, 90% power according to results of lateral distal femoral cartilage thickness, and 91% power according to the results of strain ratio of medial distal femoral cartilage (for all analyses: α = 0.05).

## 3. Results

A total of 88 volunteers (71 M and 17 F) aged 18–56 (mean 35.9 ± 8.0) were evaluated in the study. Smoking (*N* = 43) and non-smoking (*N* = 45) groups were similar in terms of age, BMI, sex, dominant extremity, mean resting knee pain scoring, and Tegner activity level (Table 1). There was no activity pain in any of the subjects. Mean amount of smoking was 10.3 ± 8.9 (range 1–45) packets/year.

High intraobserver reliability was detected for both distal femoral cartilage thickness and strain ratio measurements (Table 2). Sonographic evaluations of the subjects are given in Table 3. While cartilage thicknesses were higher in the smoking group at three sites, only the medial cartilage strain ratio measurements were found to be lower in the smoking group (all *p* < 0.05).

Correlations between smoking and sonographic evaluations are summarized in Table 4. Smoking amount was positively correlated with medial, intercondylar, and lateral cartilage thicknesses and negatively correlated medial cartilage strain ratio measurements (all *p* < 0.05).

Although the number of females was small in our study group, volunteers were further sub-grouped according to genders and non-parametric statistical analyses were performed. Medial distal femoral cartilage thicknesses and lateral distal femoral cartilage thicknesses were significantly higher (*p* = 0.015 and *p* = 0.016, respectively) in smoking men (N:35, mean thickness: 2.6 ± 0.38 and 2.3 ± 0.34, respectively) than in nonsmoking men (N:36, mean thickness: 2.3 ± 0.39 and 2.2 ± 0.29, respectively). Medial distal femoral cartilage thicknesses and intercondylar distal femoral cartilage thicknesses were significantly higher (*p* = 0.036 and *p* = 0.002, respectively) in smoking women (N:8, mean thickness: 2.3 ± 0.31 and 2.6 ± 0.35, respectively) than in nonsmoking women (N:9, mean thickness: 1.96 ± 0.32 and 2.05 ± 0.30, respectively). There was no statistically significant difference in strain ratio measurements neither between smoking and nonsmoking men nor between smoking and nonsmoking women (*p* > 0.05).

The median age of the volunteers was 37 years in our study group. We also further sub-grouped volunteers being 36 years of age and younger, and 37 years of age and older. Smokers and non-smokers compared in these age groups in terms of measured variables. Medial distal femoral cartilage thicknesses were significantly higher (*p* = 0.039) in smokers (N:24, mean thickness: 2.51 ± 0.33) than in nonsmokers (N:18, mean thickness: 2.25 ± 0.33) in 36 years of age and younger volunteers. Medial distal femoral cartilage and lateral distal femoral cartilage thicknesses were significantly higher (*p* = 0.047 and *p* = 0.001, respectively) in smokers (N:19, mean thickness: 2.55 ± 0.44 and 2.46 ± 0.34, respectively) than in nonsmokers (N:27, mean thickness: 2.27 ± 0.46 and 2.13 ± 0.32, respectively) in 37 years of age and older volunteers. There was no statistically significant difference in strain ratio measurements between smoking and nonsmoking volunteers in either group (*p* > 0.05).

## 4. Discussion

In this study, we aimed to explore whether a thorough sonographic assessment of the distal femoral cartilage would yield different results among smoking and non-smoking subjects. We have observed that the medial, intercondylar, and lateral cartilage thicknesses were higher, and that the medial cartilage strain ratio measurements were decreased in the smoking group. Further, amount of smoking was positively correlated with cartilage thickness (medial, intercondylar, and lateral) and negatively correlated with medial cartilage strain ratio (*p* < 0.05). 

Data regarding the effects of smoking on knee cartilage are controversial [7,20,21,22]. Mnatzaganian *et al.* [22] reported that dose-response relationship between smoking and total hip or knee replacement in 54,288 elderly men and women and suggested that there was an inverse association between duration of smoking and risk of undergoing total joint replacement. In a two-year prospective study of 271 smokers, Davies-Tuck *et al.* [20] reported increased tibial and patellar cartilage loss on magnetic resonance imaging (MRI). The subjects were aged between 27 and 75 (mean 58.2 ± 5.8) in their study. On the contrary, our results showed thicker femoral cartilage in smokers whereby the amount of smoking seemed to correlate positively with the thickness. Of note, our subjects did not have any knee symptoms and were younger (aged 18–56 years, mean 35.9 ± 8.0). In a recent study by Gu *et al.* [7], nicotine was shown to prevent cartilage degradation in an early osteoarthritis rat model by reducing of the serum level of TNF-α and the expression of TNF-α in the synovial tissue, and by increasing in the expression of α7nAChR in the synovial tissue. Gullahorn *et al.* [21] reported that physiologic levels of nicotine—in average level cigarette smokers—stimulated collagen and protein production in chondrocytes; however, they also reported that higher serum levels of nicotine had an inhibitor effect on chondrocytes. In this sense, we imply that our finding of thicker cartilage levels in smokers would be noteworthy. Herewith, due to the lack of TNF-α measurements in our study, we are not able to speculate on the possible underlying mechanism.

Evaluation of cartilage with real time elastography has been reported as a simple method to differentiate healthy and pathologic cartilage [15]. Cay *et al.* [15] compared strain ratios of 25 patients diagnosed as having femoral cartilage pathology on MRI sections with 25 cases diagnosed as having intact cartilage on MRI sections, and they reported that elastosonography might be an effective tool to demonstrate pathologic cartilage. To the best of the authors’ knowledge, our study is the first to evaluate the effect of smoking on sonoelastographic features of distal femoral cartilage by means of strain ratios. We observed that strain ratio measurements of the medial cartilage were lower in smokers and were negatively correlated with the amount of smoking. Considering the fact that knee osteoarthritis usually begins in the medial compartment, thicker but less elastic knee cartilage of smokers deserves attention and may indicate intrinsic changes of the cartilage architecture. Previously reported divergent effects of nicotine on collagen and glycose–amino–glycan synthesis may actually support this hypothesis [7,23,24]. Overall, whether these cartilage changes prevent or are predisposed to osteoarthritis should be investigated with further histologic studies.

We did not measure serum or synovial fluid levels of cytokines that have been previously defined to interfere with cartilage degradation. Likewise, having not measured the levels of nicotine or other toxic substances related to cigarette smoking might be considered a limitation to effectively interpret our results. One of the major limitations of our study was the cross-sectional study design. Since the cross-sectional studies can not infer causality, there is a need for prospective randomized studies to detect the causality relationship between smoking and cartilage changes. Another limitation of this study is that the results are less representative of females because of the small number of females. In addition, the ages of the volunteers were between 18 and 56 years old. Older volunteers in whom the degenerative cartilage changes and the related symptoms might be prominent were not included. Therefore, future studies are needed, including a wider population of volunteers from different age and gender groups to evaluate elastographic features of the distal femoral cartilage. Apart from the effects of smoking on the changes of cartilage, we did not evaluate various socioeconomic factors and comorbidities that may deteriorate cartilage structure, which might be considered yet another limitation of the study.

## 5. Conclusions

In light of our first and preliminary results, we imply that femoral cartilage is thicker in smokers but has less strain ratio representing harder cartilage on the medial side. Since these findings weakly correlate with the amount of smoking, and these changes could not be attributed solely to smoking, future (histologic) studies are definitely awaited to better understand how these structural changes in the knee cartilage should be interpreted with regard to the development of knee osteoarthritis in smokers.

## Figures and Tables

**Table 1:** Demographic features of the subjects.

	Nonsmokers (*N* = 45)	Smokers (*N* =43)	*p*
Age (years)	37.0 ± 7.9 (18–52)	34.7 ± 8.0 (22–56)	0.181
BMI (kg/m^2^)	26.6 ± 3.7 (19.0–34.7)	25.4 ± 4.4 (17.0–39.4)	0.157
Rest pain (VAS)	0	0.1 ± 0.6 (0–4)	0.323
**Gender**			**0.868**
M	36 (80%)	35 (81.4%)	
F	9 (20%)	8 (18.6%)	
**Dominant extremity**			**0.152**
R	44 (97.8%)	39 (90.7%)	
L	1 (2.2%)	4 (9.3%)	
**Tegner Activity Score**			**0.200**
1	1 (2.2%)	1 (2.3%)	
2	12 (26.7%)	4 (9.3%)	
3	20 (44.4%)	25 (58.1%)	
4	8 (17.6%)	6 (14%)	
5	2 (4.4%)	6 (14%)	
6	1 (2.2%)	0	
7	0	1 (2.3%)	
8	0	0	
9	1 (2.2%)	0	

**Table 2:** Intrarater reliability of cartilage thickness and strain ratio measurements.

Measurements	*p*	ICC (Lower-Upper Boundaries)
Thickness of Medial Cartilage	0.001	0.990 (0.984–0.993)
Thickness of Intercondylar Cartilage	0.001	0.992 (0.988–0.995)
Thickness of Lateral Cartilage	0.001	0.989 (0.982–0.992)
Strain Ratio of Medial Cartilage	0.001	0.965 (0.946–0.997)
Strain Ratio of Intercondylar Cartilage	0.001	0.928 (0.890–0.953)
Strain Ratio of Lateral Cartilage	0.001	0.958 (0.935–0.972)

**Table 3:** Cartilage thickness (mm) and strain ratio measurements (mean ± SD).

Measurements	Nonsmokers (*N* = 45)	Smokers (*N* = 43)	*p*
Thickness *(medial)*	2.27 ± 0.41	2.53 ± 0.38	0.002
Thickness *(intercondylar)*	2.41 ± 0.49	2.63 ± 0.35	0.017
Thickness *(lateral)*	2.15 ± 0.31	2.35 ± 0.33	0.004
Strain ratio *(medial)*	0.24 ± 0.12	0.18 ± 0.06	0.003
Strain ratio *(intercondylar)*	0.19 ± 0.07	0.17 ± 0.05	0.050
Strain ratio *(lateral)*	0.17 ± 0.05	0.15 ± 0.06	0.231

**Table 4:** Correlations between amount of smoking and sonographic measurements.

Measurements	*p*	r
Medial cartilage thickness	0.045	0.214
Intercondylar cartilage thickness	0.001	0.378
Lateral cartilage thickness	0.003	0.316
Medial cartilage strain ratio	0.034	−0.226
Intercondylar cartilage strain ratio	0.344	−0.102
Lateral cartilage strain ratio	0.924	0.010

## Abbreviations

The following abbreviations are used in this manuscript:

US: Ultrasonography/ultrasound
ROI: Region of interest
BMI: Body mass index
VAS: Visual analog scale
ICC: Interclass correlation coefficient
MRI: Magnetic resonance imaging
